# Supplementary figures and images for: Molecular screening and dynamics simulation reveal potential phytocompounds in Swertia chirayita targeting the UspA1 protein of Moraxella catarrhalis for COPD therapy
Source: PLoS One. 2025 Feb 28;20(2):e0316275. doi: 10.1371/journal.pone.0316275 (PMC11870343; doi:10.1371/journal.pone.0316275)

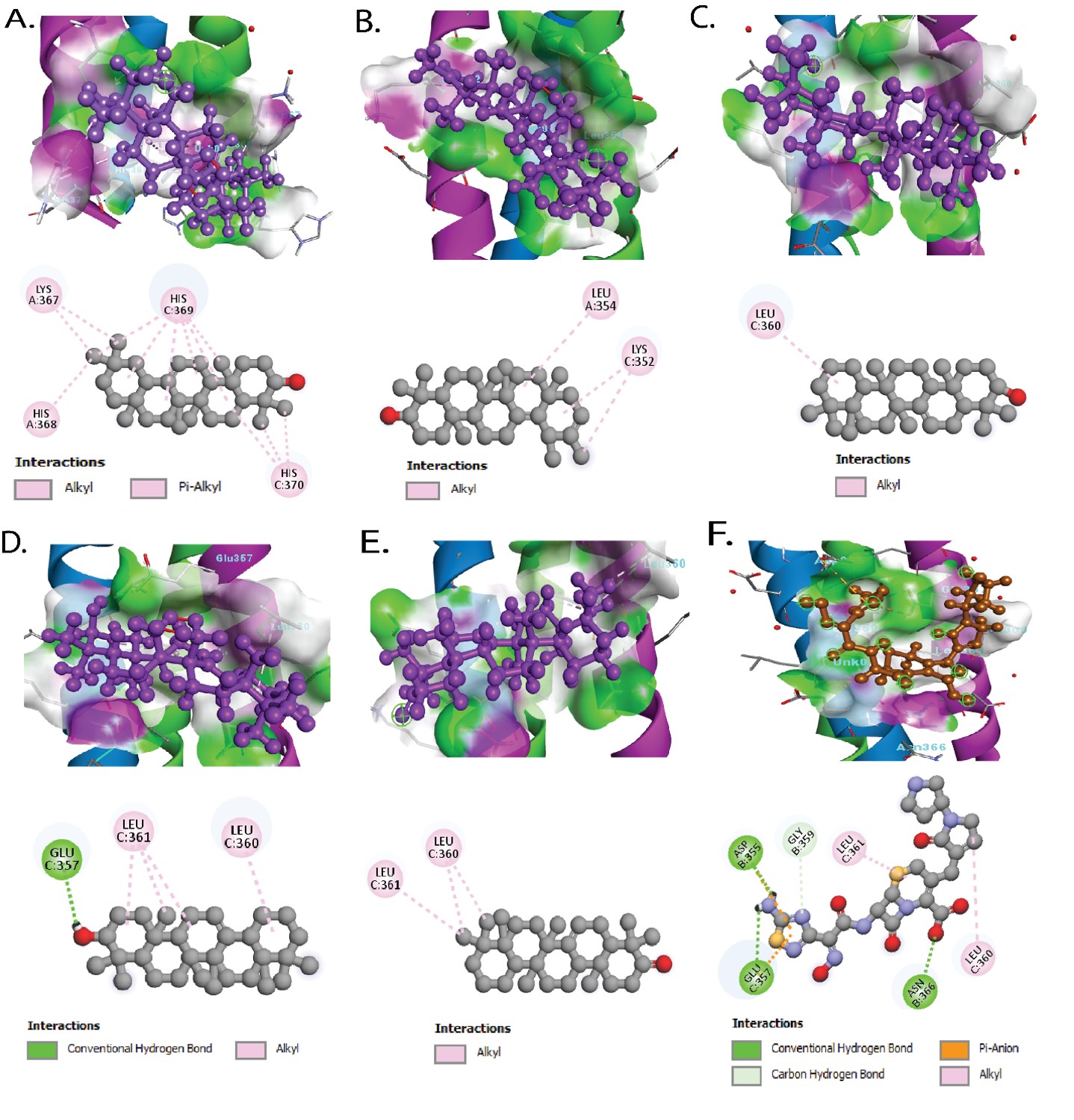

Supplement: S1 Fig — On upper panel, (A) CEACAM1 and beta-amyrin, (B) CEACAM1 and calendol, (C) CEACAM1 and episwertenol, (D) CEACAM1 and kairatenol, and (E) CEACAM1 and swertanone. The lower panel shows the three-dimensional structure of all complexes. (JPG) [file pone.0316275.s001.jpg]
