# Supplementary material for: Molecular screening and dynamics simulation reveal potential phytocompounds in Swertia chirayita targeting the UspA1 protein of Moraxella catarrhalis for COPD therapy
Source: PLoS One. 2025 Feb 28;20(2):e0316275. doi: 10.1371/journal.pone.0316275 (PMC11870343; doi:10.1371/journal.pone.0316275)
Supplement: Attachment — Submitted filename: Comments of Manuscript Number PONE-D-24-44469.docx [file pone.0316275.s002.docx]

**Comments:**

1. Introduction: There is redundancy in discussing *M. catarrhalis*’s role in COPD exacerbations, AMR challenges, and traditional medicine. Consolidating these points will improve clarity and reduce length.

2. Introduction: Topics such as bacterial infections, AMR, traditional medicine, and *S. chirayita* are presented in fragmented sections. To enhance coherence, consider reordering the introduction to move from COPD challenges and bacterial contributions to exacerbations, then to the role of *M. catarrhalis*, and finally to *S. chirayita* as a potential traditional treatment.

3. Introduction: Computational approaches like molecular docking and dynamics are introduced without detailing their relevance. A brief explanation of each method (e.g., docking for binding affinity and MDS for stability) would clarify their specific contributions to UspA1 inhibition analysis.

4. Introduction: Extensive detail on *S. chirayita*’s general uses is provided, but it lacks specificity on its potential against *M. catarrhalis*. Highlighting antimicrobial properties relevant to respiratory pathogens would enhance focus on COPD management.

5. Results: Docking scores are promising for UspA1, yet lack comparative context. Including baseline values for known UspA1 inhibitors would emphasize the efficacy of the tested compounds. Additionally, elaborating on key amino acid interactions could clarify why certain residues enhance binding stability, especially compared to the control drug, ceftobiprole.

6. Molecular Dynamics Simulation (MDS): Including a brief explanation of why an RMSD above 1.5 Å might be acceptable for certain protein-ligand complexes would add clarity.

7. Binding Free Energy and MM/GBSA Analysis: While the MM/GBSA results provide insights into binding energy, they could be contextualized by comparing to experimental values where available. Including binding energy snapshots throughout the simulation could further substantiate claims of interaction stability for beta-amyrin and kairatenol.

8. A brief discussion on the therapeutic relevance of targeting UspA1 in *M. catarrhalis* and the physiological role of CEACAM1 in COPD treatment would add value, particularly on how observed interactions may impact COPD pathogenesis or immune modulation.

9. Discussing the limitations of in-silico approaches alone would improve the manuscript’s credibility. Acknowledging challenges in ADME/toxicity predictions without in vitro validation would demonstrate a balanced perspective.

10. While UspA1’s role in *M. catarrhalis* pathogenicity is well-covered, CEACAM1’s relevance to COPD or bacterial infections is unclear. Expanding on its role in immune response or as a therapeutic target would improve the rationale for its inclusion.

11. Table 1: There should be “hydrophobic interactions” instead of “hydrophobic bond”.

12. Unit of the docking grid whether it is ‘Å’ or ‘nm’ should be clearly mentioned. Also, provide dimension of the grid center.
